# Angiomotin promotes renal epithelial and carcinoma cell proliferation by retaining the nuclear YAP

**DOI:** 10.18632/oncotarget.7161

**Published:** 2016-02-03

**Authors:** Meng Lv, Shuting Li, Changqin Luo, Xiaoman Zhang, Yanwei Shen, YanXia Sui, Fan Wang, Xin Wang, Jiao Yang, Peijun Liu, Jin Yang

**Affiliations:** ^1^ Department of Medical Oncology, The First Affiliated Hospital of Xian Jiaotong University, Xi'an, Shaanxi 710061, P.R. China; ^2^ Center for Translational Medicine, The First Affiliated Hospital of Xian Jiaotong University, Xi'an, Shaanxi 710061, P.R. China; ^3^ Department of Pathology, The First Affiliated Hospital of Xi'an Jiaotong University, Xi'an, Shaanxi, 710061, P.R. China; ^4^ Department of Gastroenterology, The Central Hospital of Ankang City, Ankang, Shaanxi 725000, P.R. China; ^5^ Department of Oncology, Shangluo Central Hospital, Shangluo, Shaanxi, 726000, P.R. China

**Keywords:** Angiomotin, renal epithelial cells, renal cell carcinoma, proliferation, YAP

## Abstract

Renal cell carcinoma (RCC) is one of the common tumors in the urinary system without effective therapies. Angiomotin (Amot) can interact with Yes-associated protein (YAP) to either stimulate or inhibit YAP activity, playing a potential role in cell proliferation. However, the role of Amot in regulating the proliferation of renal epithelial and RCC cells is unknown. Here, we show that Amot is expressed predominantly in the nucleus of RCC cells and tissues, and in the cytoplasm and nucleus of renal epithelial cells and paracancerous tissues. Furthermore, Amot silencing inhibited proliferation of HK-2 and 786-O cells while Amot upregulation promoted proliferation of ACHN cells. Interestingly, the location of Amot and YAP in RCC clinical samples and cells was similar. Amot interacted with YAP in HK-2 and 786-O cells, particularly in the nucleus. Moreover, Amot silencing mitigated the levels of nuclear YAP in HK-2 and 786-O cells and reduced YAP-related CTGF and Cyr61 expression in 786-O cells. Amot upregulation slightly increased the nuclear YAP and YAP-related gene expression in ACHN cells. Finally, enhanced YAP expression restored proliferation of Amot-silencing 786-O cells. Together, these data indicate that Amot is crucial for the maintenance of nuclear YAP to promote renal epithelial and RCC proliferation.

## INTRODUCTION

Renal cell carcinoma (RCC) is one of the common malignant tumors in the urinary system [1]. Its incidence is increasing in the world, including in China [2-3]. Currently, treatment of patients with RCC depends on surgery, which is not suitable for patients with metastatic RCC [4]. Hence, understanding the pathogenic process and discovering new targets are crucial for development of effective therapies.

The Hippo signal pathway is involved in an evolutionarily conserved kinase cascade and regulates cell fate determination, including tumorigenesis [5]. Yes-associated protein (YAP) and transcriptional co-activator with PDZ-binding motif (TAZ), two key downstream transcription co-activators, can bind to several transcription factors, such as TEADs, and promote tumor cell proliferation [6-7]. Indeed, high levels of YAP/TAZ have been detected in patients with different types of cancers, including RCC [8-11]. The YAP and TAZ have been considered as oncogenes and down-regulation of YAP/TAZ may be valuable for inhibition of RCC progression. Notably, Angiomotin (Amot) is a member of the motin family of angiostatin binding proteins and contains conservative coiled-coil domains and C-terminal PDZ binding motifs, regulating the migration, angiogenesis and endothelial cell function [12-14]. There are three members in the Amot family: Amot (p80 and p130 isoforms), Amot-like protein 1 (AmotL1) and Amot-like protein 2 (AmotL2). Amot p130, AmotL1, and AmotL2 contain conservative glutamine-rich domains and PPxY motifs in their N-terminus, but Amot-p80 lacks the entire N-terminal [15]. The function of Amot family members in regulating cell proliferation appears to be controversial and is tissue and cell type-specific [16-21]. While the Amot family members can inhibit the proliferation of non-tumor kidney epithelial MDCK cells and human embryonic kidney (HEK) 293 cells by inhibiting YAP [17-18], other studies indicate that Amot can act as a co-activator of YAP to promote the growth of hepatocarcinoma cells and breast cancer [19, 21]. In addition, a previous study has shown that translocation of Amot-p130-YAP complex into the nucleus promotes the transcription of TEAD-target genes while other studies have reported that phosphorylation of Amot by LATS promotes Amot-YAP association in the cytoplasm and subsequently inhibits YAP activity [15]. However, the role of Amot/YAP in regulating RCC proliferation has not been explored.

In this study, we investigated the expression pattern of Amot/YAP in RCC and examined the regulatory effect of Amot/YAP on the proliferation of RCC cells as well as the potential molecular mechanisms.

## RESULTS

### The distribution of Amot expression in renal tubular epithelial cells, RCC cells, RCC tissues and para-cancerous tissues

**Figure 1:**
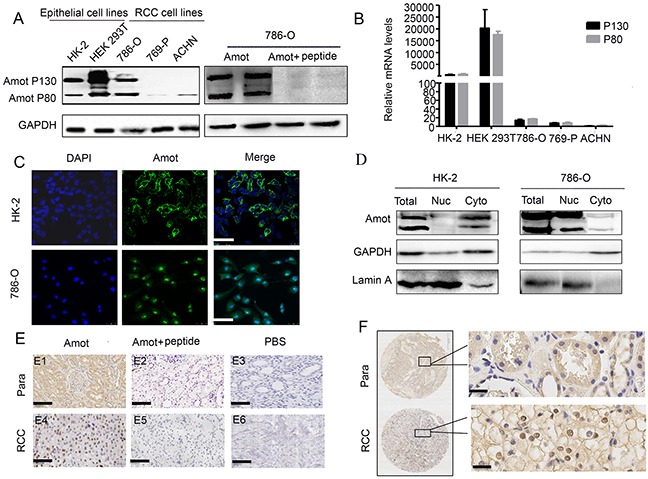
The distribution of Amot in renal epithelial cells and RCC cells **A.** Western blot analysis of the relative levels of Amot expression in different epithelial and RCC cell lines (left panel). The specificity of anti-Amot in 786-O cells was analyzed by Western blot (right panel). **B.** Quantitative RT-PCR analysis of the relative levels of Amot mRNA transcripts in the different epithelial and RCC cell lines. **C.** Immunofluorescent analysis of Amot expression in HK-2 and 786-O cells. Scale bar = 25 μm. **D.** Western blot analysis of the distribution of cytoplasmic and nuclear Amot in HK-2 and 786-O cells (Nuc: nucleus; Cyto: cytoplasm). Lamin A was used as a nuclear marker. **E.** Immunohistochemsitry of Amot expression in human RCC and paracancerous tissues (Para: paracancerous tissues). E1, E4: anti-Amot; E2, E5: anti-Amot and antigen peptides; E3, E6: PBS control; Scale bar = 146 μm. **F.** Immunohistochemistry of Amot expression in human RCC and paracancerous tissues. Scale = 33μm.

To characterize the expression pattern of Amot, the expression of Amot in different renal cells (RCC 786-O, 769-P, ACHN, non-tumor renal epithelial HK-2 and HEK 293T) was determined by Western blot and RT-PCR assays. High levels of Amot p130 and p80 expression were detected in HK-2, HEK 293T and 786-O cells and only a little Amot p80 was detected in 769-P and ACHN cells (Figure 1A and 1B). Immunofluorescence assay revealed that the Amot expression was predominantly located in the cytoplasm of HK-2 cells, but in the nucleus of 786-O cells (Figure 1C). Similarly, the differential distribution of Amot between HK-2 and 786-O cells was further demonstrated by Western blot (Figure 1D). Furthermore, we characterized the Amot expression pattern in 75 RCC and paracancerous tissues and found that Amot expression was detected in 52 RCC and 45 paracancerous tissues. The Amot expression was predominantly detected in the nucleus of RCC tissues (47/52), but in the cytoplasm of the paracancerous tissues (26/45, Figure 1E and 1F). In other RCC (5/52) and paracancerous tissues (19/45), Amot was detected in the nucleus and cytoplasm. The results were consistent with the findings from cell lines. Hence, the distribution of Amot in RCC was different from that in non-tumor renal epithelial cells.

### The distribution of Amot in renal tubular epithelial cells, but not RCC cells, is associated with cell density

**Figure 2:**
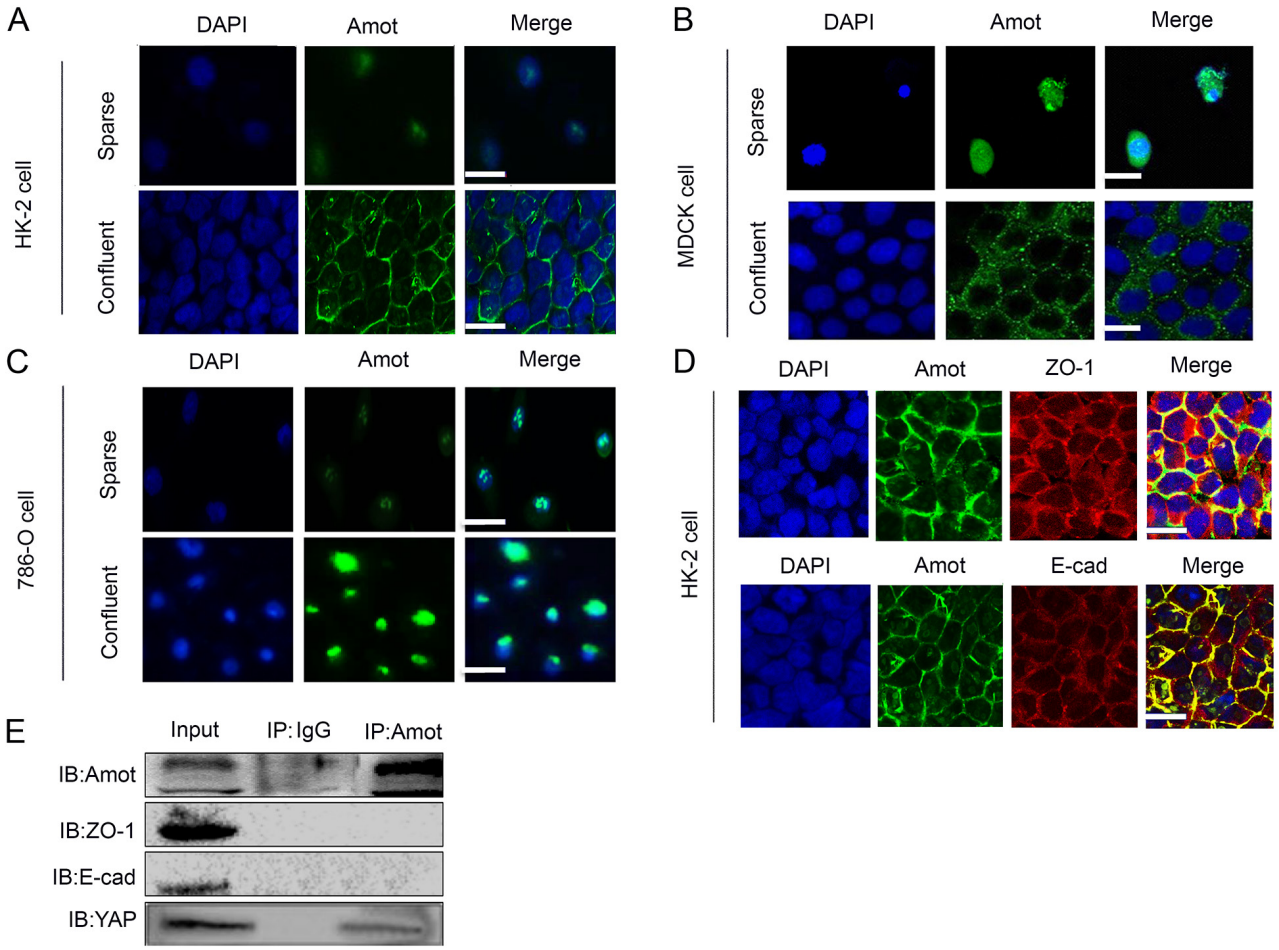
The distribution of Amot depends on cell density in renal epithelial cells, but not in RCC cells **A, B.** Immunofluorescent analysis of Amot in HK-2 and MDCK cells cultured in sparse and confluent density. Sparse: 30% density, confluent: 100% density; Scale bar = 25 μm. **C.** Immunofluorescence of Amot in 786-O cells cultured in sparse and confluent density. Scale bar = 15 μm. **D.** Immunofluorescent analysis of Amot and ZO-1 or E-cadherin in HK-2 cells. Scale bar = 25 μm. **E.** Immunoprecipitation (IP) with the anti-Amot antibody and Western blotting analysis of Amot, ZO-1 and E-cadherin interaction in HK-2 cells. Rabbit IgG was used as a negative control and anti-YAP as a positive control.

To understand the mechanisms underlying the different distribution of Amot between non-tumor renal epithelial and RCC cells, HK-2 and 786-O cells were cultured in different densities and their Amot expression was characterized by immunofluorescence. First, the Amot was detected predominantly in the nucleus of HK-2 cells cultured in a sparse density and detected in the cytoplasm and membrane of confluent HK-2 cells (Figure 2A). These results suggested that the Amot migrated from the nucleus to cytoplasm of HK-2 cells with increased cell density. A similar pattern of Amot expression was detected in non-tumor MDCK cells (Figure 2B). In contrast, the Amot was exclusively detected in the nucleus of 786-O cells regardless the cell density (Figure 2C and Supplementary Figure S1). Moreover, we found that the Amot expression was co-localized with ZO-1, a marker for cell junctions, and E-cadherin, a marker of cell adherens junctions, in confluent HK-2 cells (Figure 2D). However, immunoprecipitation assay failed to reveal any direct interaction between Amot and ZO-1 or E-cadherin (Figure 2E), consistent with a previous report in MDCK cells [14]. These data suggest that the distribution of Amot in renal epithelial cells, but not RCC cells, may be associated with their cell density and the Amot may play different functions in non-tumor and RCC cells.

### Amot promotes the proliferation of renal epithelial and RCC cells

**Figure 3:**
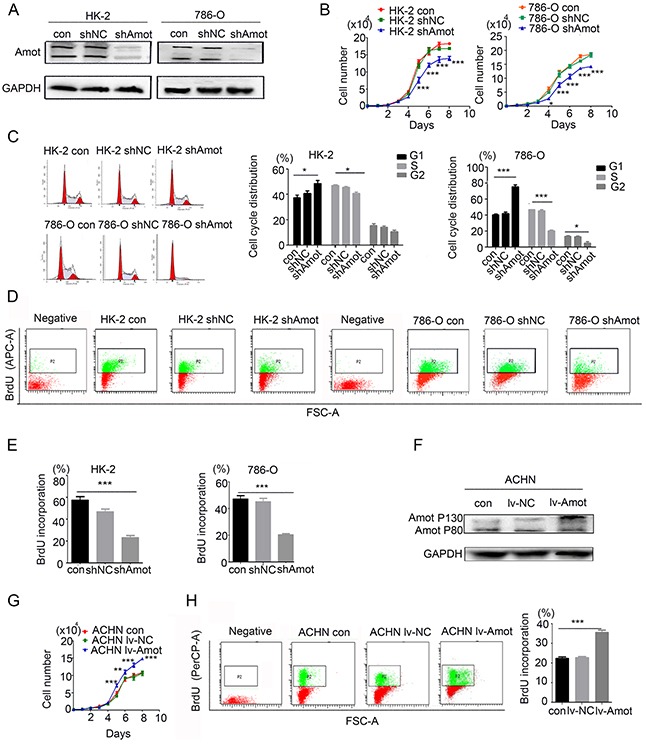
Amot promotes proliferation of renal epithelial and RCC cells **A.** Western blot analysis of Amot silencing in HK-2 and 786-O cells. **B.** Knockdown of Amot inhibits HK-2 and 786-O cell proliferation. The shAmot group of cells was compared with control group or shNC group of cells. **C.** Knockdown of Amot induces cell cycle arrest at G1 phase in HK-2 and 786-O cells. **D, E.** Knockdown of Amot inhibits BrdU incorporation in HK-2 and 786-O cells using APC-anti-BrdU. Negative: The cells were treated with PBS in place of Anti-BrdU antibody as the negative control. **F.** Western blot analysis of up-regulated Amot p130 expression in ACHN/lv-Amot cells. **G.** Enhanced Amot expression promotes ACHN cell proliferation. The lv-Amot group of cells was compared with the control group or lv-NC group of cells. **H.** Up-regulated Amot expression enhances BrdU incorporation in ACHN/lv-Amot cells using PerCP-Anti-BrdU. Negative: The cells were treated with PBS in place of Anti-BrdU antibody as the negative control. Con: Unmanipulated cells; NC (Negative control): the cells infected with control lentivirus; shAmot (Amot knockdown); The cells infected with shAmot lentivirus; lv-Amot (Up-regulated Amot expression): The cells infected with lv-Amot lentivirus. Data are representative images and expressed as the mean ± SD of each group of cells from three separate experiments. *P < 0.05; **P < 0.01; ***P < 0.001.

**Figure 4:**
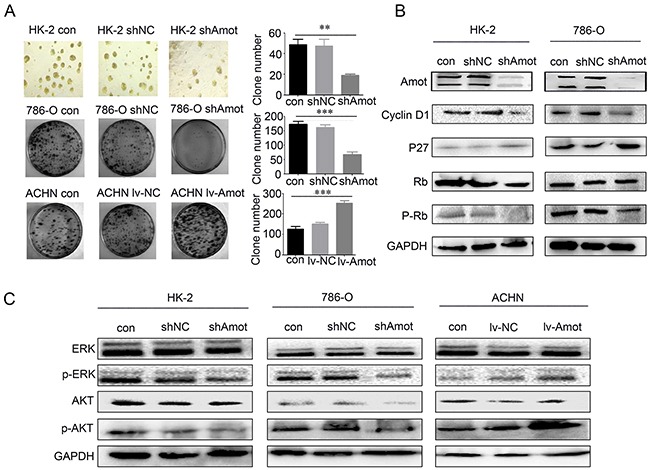
Amot promotes clone formation and regulates proliferation-related signaling in renal epithelial and RCC cells **A.** Amot promotes clone formation in renal epithelial(three-dimensional cell culture) and RCC cells (plate clone formation assay). **B.** Western blot analysis of cell cycling-related regulators. **C.** Western blot analysis of the relative levels of ERK and AKT expression and phosphorylation in renal epithelial and RCC cells. Data are representative images and expressed as the mean ± SD of each group of cells from three separate experiments. GAPDH was used as an internal control.

To investigate the function of Amot, we tested whether modulation of Amot expression could change the proliferation of renal epithelial and RCC cells. We first generated stably Amot silencing RCC cells (786-O/shAmot), renal tubular epithelial cells (HK-2/shAmot), up-regulated Amotexpression RCC cells (ACHN/lv-Amot) and relevant controls. Furthermore, we characterized the relative levels of Amot expression by Western blot assays and found significantly reduced levels of Amot expression in HK-2/shAmot and 768-O/shAmot cells while up-regulated Amot expression in ACHN/lv-Amot cells, related to that in the controls (Figure 3A and 3F). Subsequently, we detected significantly reduced proliferation of HK-2/shAmot and 768-O/shAmot cells while enhanced logarithmic proliferation of ACNH/Lv-Amot cells (Figure 3B and 3G). To understand the mechanisms underlying the action of Amot, we analyzed the cell cycling and BrdU incorporation by flow cytometry. In comparison with that in the shNC controls, knockdown of Amot expression induced cell cycle arrest at G1 phase and reduced BrdU incorporation in both HK-2/shAmot and 768-O/shAmot cells while up-regulated Amot expression enhanced BrdU incorporation in ACHN/lv-Amot cells in vitro (Figure 3C–3H). Moreover, we analyzed the capacity of different groups of cells to form clones in 3-d cultures and plate colony, and found that knockdown of Amot significantly reduced the numbers of clones in HK-2/shAmot and 768-O/shAmot cells, but up-regulated Amot expression significantly increased the numbers of clones in ACHN/lv-Amot cells (Figure 4A). These data clearly indicated that Amot promoted renal epithelial and RCC cell proliferation in vitro.

Further analyses revealed that knockdown of Amot significantly reduced the relative levels of cyclin D1 expression and retinoblastoma (Rb), ERK and AKT phosphorylation, but significantly increased the levels of p27 expression in HK-2/shAmot and 768-O/shAmot cells (Figure 4B and 4C). In contrast, up-regulated Amot expression enhanced the relative levels of ERK and AKT phosphorylation in ACHN/lv-Amot cells (Figure 4C). Therefore, Amot may enhance cell cycling by up-regulating cyclin D1 expression and enhancing the ERK and AKT signaling, promoting the proliferation of renal epithelial and RCC cells.

### Amot is crucial for nuclear accumulation and activity of YAP in RCC cells

**Figure 5:**
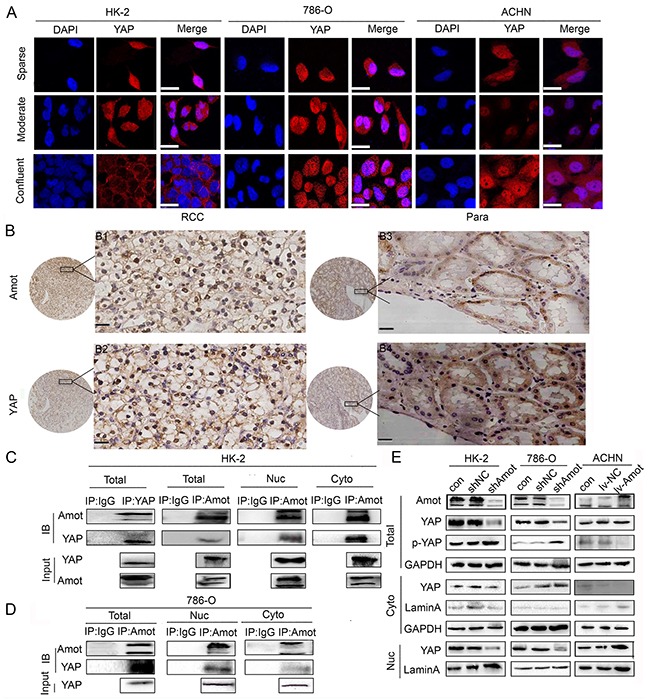
Amot is crucial for retaining the nuclear YAP **A.** Immunofluorescence of Amot in HK-2,786-O and ACHN cells cultured at sparse, moderate and confluent density. Sparse: 30% density, moderate: 60%-80% density, confluent: 100% density; Scale bar = 25μm **B.** Immunohistochemistry of Amot and YAP expression in human RCC and paracancerous tissues. Para: paracancerous tissues; B1, B3: anti-Amot; B2, B4: anti-YAP; Scale = 33μm. **C.** Immunoprecipitation and Western blotting analysis of endogenous Amot-YAP complexes in HK-2 cells (IB). Western blot analysis of Amot and YAP in cell lysates from HK-2 cells (Input). Rabbit IgG from unvaccinated animals was used as the control. **D.** Immunoprecipitation and Western blotting analysis of endogenous Amot-YAP complexes in 786-O cells (IB). Western blot analysis of Amot and YAP in cell lysates from 786-O cells (Input). **E.** Western blot analysis of Amot, total YAP, phosphorylated YAP, GAPDH, and Lamin A in cytoplasmic and nuclear fractions of cells. GAPDH was used as an internal control. Lamin A was used as a nuclear marker.

**Figure 6:**
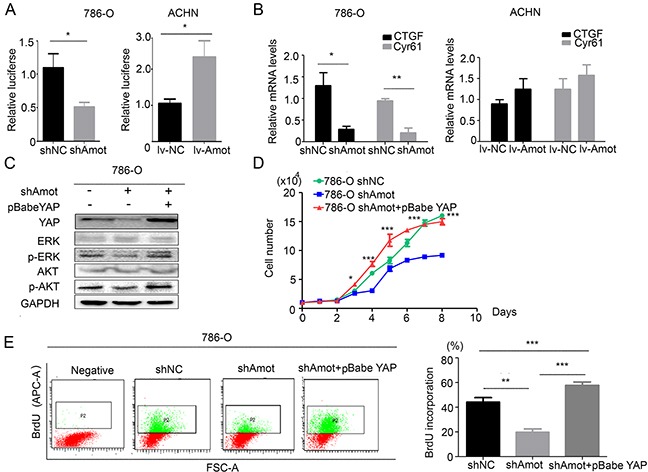
Amot is crucial for YAP activity in RCC cells **A.** Luciferase reporter assays indicate that Amot enhances the YAP-related TEAD promoter activity in RCC cells. **B.** Quantitative RT-PCR reveals that Amot is important for YAP-related CTGF and CYR61 mRNA transcription in RCC cells. **C.** Western blot analysis of YAP, ERK, AKT phosphorylation and expression. **D.** Enhanced YAP expression restores proliferation of Amot-silencing 786-O cells. The shAmot+pBabe YAP group was compared with the shAmot group. **E.** Enhanced YAP expression restores BrdU incorporation in Amot-silencing 786-O cells. The shAmot+pBabe YAP group: shAmot cells were transfected with pBabe YAP plasmid to induce YAP overexpression. Data are representative images, charts and expressed as the mean ± SD of each group of cells from three separate experiments. *P<0.05, **P<0.01, ***P<0.001.

Amot can bind to and retain nuclear YAP, leading to the activation of downstream signaling. To understand the relationship of Amot and YAP in RCC and renal epithelial cells, we characterized the cytoplasmic and nuclear YAP in different groups of cells cultured in varying densities by immunofluorescence assay. We detected YAP in the nucleus of sparse HK-2 cells and in the cytoplasm and nucleus of moderate density of HK-2 cells, but predominantly in the membrane of confluent HK-2 cells. In contrast, we observed that YAP was present in the cytoplasm and nucleus of 786-O and ACHN cells regardless of cell density (Figure 5A). Immunofluorescence assay showed that the location of YAP was similar to Amot in HK-2 cells and 786-O cells (Figures 2A and 2C). Moreover, we examined the expression of YAP and Amot in 10 pairs of RCC and paracancerous tissues. Firstly, we randomly selected and examined 2 pairs of RCC samples and paracancerous tissues from the same patient. We noticed that YAP expression was predominantly detected in the nucleus of RCC tissues, but in the cytoplasm of the paracancerous tissues, consistently with the Amot expression (Figure 5B). Next we examined the expression of Amot and YAP in another 9 RCC patients’ tissues and found that in most of the patient's tissues (7/10), the location of YAP and Amot in RCC tissues and paracancerous tissues was similar. Next, we explored the relationship of Amot and YAP in HK-2 and 786-O cells by immunoprecipitation assay. The results indicated that antibodies against Amot efficiently pulled down cytoplasmic and nuclear YAP in HK-2 and 768-O cells (Figures 5C and 5D). The relative levels of nuclear YAP precipitated by anti-Amot were obviously higher than that of cytoplasmic YAP from 768-O cells, indicating a close interaction of Amot with YAP in the nucleus of RCC. In addition, knockdown of Amot significantly reduced the levels of cytoplasmic and nuclear YAP, particularly in the nucleus, but remarkably increased the relative levels of YAP phosphorylation in both HK-2/shAmot and 768-O/shAmot cells (Figure 5E). However, up-regulated Amot expression increased the relative levels of nuclear YAP, but decreased the relative levels of cytoplasmic YAP and YAP phosphorylation in ACHN/Lv-Amot cells (Figure 5E). These data indicated that Amot was crucial for the nuclear accumulation of YAP in renal epithelial and RCC cells and for enhancing their proliferation. Finally, we examined the role of Amot in regulating YAP function in RCC cells. We employed dual luciferase assay and found that knockdown of Amot significantly decreased the transcription activity of the YAP-related TEAD promoter activity in 786-O cells while enhanced Amot expression significantly increased the YAP-related TEAD promoter activity in ACHN cells (Figure 6A). Furthermore, knockdown of Amot significantly decreased the relative levels of YAP-targeted CTGF and Cyr61 mRNA transcripts in 768-O/shAmot cells while enhanced Amot expression slightly increased the relative levels of CTGF and Cyr61 mRNA transcripts in ACHN/lv-Amot cells (Figure 6B). Interestingly, transfection with pBabe YAP plasmid up-regulated YAP expression, particularly in the nuclear YAP accumulation, and enhanced proliferation, BrdU incorporation, relative levels of ERK and AKT phosphorylation in 768-O/shAmot cells (Figure 6C-6E). Collectively, these data indicated that Amot was crucial for the nuclear YAP accumulation, YAP-mediated signaling and RCC proliferation.

## DISCUSSION

In our study, we first investigated the distribution of Amot in renal epithelial and RCC cells. We found that the distribution of Amot was dependent on the cell density in renal epithelial cells and Amot migrated from the nucleus to cytoplasm and membrane following increased density of cells. Although Amot was co-localized with ZO-1 and E-cadherin in the membrane of confluent HK-2 and MDCK cells, Amot did not interact with ZO-1 and E-cadherin in these cells, consistent with a previous report [14]. Wells et al. [14] reported that Amot was localized to tight junctions following a Ca2+ switch, later than the retargeting of ZO-1. Accordingly, Amot may be not necessary for the formation of tight junctions, but may help stabilize it in renal epithelial cells. On the other hand, we found that Amot was localized predominantly in the nucleus of malignant RCC tissues and RCC 768-O cells regardless of cell density. Hence, our data support the notion that Amot may have different functions in different types of cells. We are interested in investigating whether altered Amot expression can change the distribution and function of Amot in renal epithelial cells.

Recent studies report that Amot regulates cell proliferation, but the role of Amot in cell proliferation is controversial [15, 19]. Knockdown of Amot promotes proliferation and migration of lung cancer cells in vitro and in vivo [22], but inhibits proliferation of breast cancer cells [21]. We found that knockdown of Amot inhibited proliferation of renal epithelial and RCC cells, accompanied by inducing cell cycle arrest at G1 phase and reducing the ERK and AKT phosphorylation. In contrast, enhanced Amot expression promoted proliferation of ACHN cells, accompanied by increased ERK and AKT phosphorylation. Our data extended previous findings [16, 19, 23-28] and indicated that Amot predominantly promoted proliferation of renal epithelial and RCC cells. Given that the ERK and AKT signaling is important for cell proliferation it is possible that Amot through interaction with YAP may enhance the ERK/AKT signaling in renal epithelial and RCC cells.

Previous studies have suggested that YAP/TAZ may be oncogenes in mammalian cells [8-11]. Furthermore, Schütte U et al [11] reported that high levels of nuclear YAP expression were detected in a subset of patients with RCC and knockdown of YAP significantly inhibited growth of clear cell RCC (ccRCC) cells in vitro and vivo. These data indicate that YAP play an important role in proliferation of ccRCC cells.

In this study, we found that the Amot and YAP had similar location in RCC tissues and cells. Interestingly, Amot was co-localized and interacted with YAP in growing renal epithelial and RCC cells and knockdown of Amot preferably reduced the levels of nuclear YAP and YAP-downstream CTGF and Cyr61 expression, but increased the levels of YAP phosphorylation in both renal epithelial and RCC cells. In contrast, enhanced Amot expression increased the nuclear YAP, CTGF and Cyr61 expression, but decreased the levels of YAP phosphorylation in ACHN cells. Furthermore, knockdown of Amot significantly reduced the YAP-related TEAD promoter activity in 786-O cells while enhanced Amot expression significantly increased the YAP-related TEAD promoter activity in ACHN cells. More importantly, we found that enhanced YAP expression restored proliferation and BrdU incorporation in Amot-silencing RCC 786-O cells. A recent study has indicated that Amot acts as a YAP cofactor, and can prevent YAP phosphorylation to enhance YAP activity [19]. Given that Amot and YAP were co-localized in the nucleus of RCC cells, our data suggest that Amot may be crucial for the maintenance of nuclear YAP to promote renal epithelial and RCC cell proliferation.

In summary, our data indicated that the distribution of Amot expression was dependent on cell density in renal epithelial cells, but predominantly in the nucleus of some RCC cells. Amot promoted proliferation of renal epithelial and RCC cells by retaining and interaction with the nuclear YAP as well as preventing YAP phosphorylation. Our findings may provide new insights into regulating the Amot/YAP-related signaling in renal epithelial and RCC cells proliferation.

## MATERIALS AND METHODS

### Antibodies

The rabbit antibodies against Amot were produced by Genemed Synthesis (San Antonio, USA) using the synthesized peptides (C-KTPIQILGQEPDAEMVEYLI) conjugated to keyhole limpet hemocyanin (KLH) for immunizations. Antibodies against GAPDH, Lamin A, cyclin D1, Rb, p-Rb as well as rabbit and mouse anti-Flag were from Santa Cruz BioTechnology; YAP, p-YAP, ERK, p-ERK, AKT, p-AKT from Cell Signaling Technology; PerCP mouse and APC mouse anti-BrdU from BD Transduction Laboratories.

### Clinical samples

The RCC and adjacent non-cancerous tissue samples were obtained from 75 RCC patients, who underwent surgical resection at the First Affiliated Hospital of Xi'an Jiaotong University. Individual patients with RCC were diagnosed by pathological examination. Written informed consent was obtained from individual patients and the experimental protocol was approved by the Ethics Committee of Xi'an Jiaotong University.

### Cell culture, generation of stable transfectants and plasmids

786-O, 769-P, ACHN and renal epithelial HK-2 and HEK 293T cells were purchased from the Cell Resource Center of Peking Union Medical College (Beijing, China). HK-2 and ACHN cells were cultured in DMEM F-12 medium with 10% fetal bovine serum (FBS, HyClone, Logan, UT) as complete medium, and other types of cells were cultured in RPMI-1640 medium supplemented with 10% FSB, penicillin (100 IU/ml) and streptomycin (0.1 mg/ml) as complete medium in 5% CO_2_ at 37°C.

Lentivirus for expression of human Amot small hairpin RNA (shRNA) were constructed, generated and purified by GeneChem (Shanghai, China). The sequences of RNA interference were shAmot (8489-1R), 5′-TGCAGAGATGGTGGAATAT-3′; shAmot (8491-2R) 5′-ACACATCGAAATCCGAGAT-3′; and control shNC: 5′-TTCTCCGAATGTGTCACGT-3′. HK-2 and 786-O cells were cultured in complete medium in six-well plates overnight and infected with lentivirus at multiplicity of infection (MOI) of 20, according to the manufacturer's instructions. Twelve hours after transfection, the cells were treated with puromycin for 3 weeks to generate stable Amot silencing cells. Similarly, ACHN cells were infected with lv-Amot lentivirus (#54796, GeneChem) at MOI of 10 to generate stably up-regulated Amot expression cells, according to the manufacturer's instructions.

The unmanipulated cells (control), cells infected with lentivirus for shNC expression (shNC) or control lentivirus (lv-NC), with lentivirus for shAmot overexpression (shAmot), or with lentivirus for Amot over expression (lv-Amot) were simultaneously tested. To induce YAP over-expression, cells were transfected with pBabe YAP plasmid (#10413, GeneChem).

### Western blot analysis

Different groups of cells were lyzed in RIPA buffer (BD PharMingen, San Diego, CA) and after being centrifuged, the cell lysates were separated by sodium dodecyl sulfate polyacrylamide gel electrophoresis (SDS-PAGE) and transferred onto polyvinyldifluoride (PVDF) membranes (0.22 μm, Millipore, Boston, MA). The membranes were blocked with 5% fat-free dry milk in TBST and incubated with primary antibodies against Yap, p-Yap, ERK, p-ERK, AKT, p-AKT, Amot, cyclin D1, pRb, Rb, GAPDH and Lamin A (1:1000 dilution) at 4°C overnight. The bound antibodies were detected with horseradish peroxidase (HRP)-conjugated secondary antibodies (1:3000, Santa Cruz BioTech, CA) and visualized using the ECL detection system (Millipore), according to the manufacturer's instructions.

### Immunohistochemistry and immunofluorescence

The tissue sections were deparaffinized, rehydrated, and subjected to antigen retrieval in EDTA buffer (1.0 mM, pH 8.0) for 10 min in a microwave oven. The sections were blocked with 10% goat sera and were incubated with primary antibodies against Amot or rabbit sera (1:1000) overnight at 4°C. After being washed, the bound antibodies were detected with HRP-conjugated secondary antibody for 30 min and visualized with 3,3-diaminobenzidine, followed by counterstained with hematoxylin. The sections were observed under a microscope (Olympus cx21, Tokyo, Japan).

Cells on coverslips were fixed by 4% paraformaldehyde and treated with 0.1% Triton for 10 minutes in the dark. The cells were blocked with 0.2% BSA and incubated with primary antibodies against Amot (1:100) overnight at 4°C. After being washed, the bound antibodies were detected with fluorescent anti-rabbit secondary antibody (1:500, Santa Cruz Biotechnology) for 2 hours and the cells were stained with 4, 6-diamino-2-phenyl indole (DAPI). Immunofluorescence was analyzed using a laser scanning confocal microscope (Leica, Germany).

### Cell growth assays

Different groups of cells (2000 cells/well) were cultured in 24-well plates. The numbers of cells in each well were quantified every day under a microscope (Leica, Germany) in a blinded manner.

### Cell cycle assay

786-O and HK-2 cells at 1 ×10^5^ cells/ml were cultured in six-well plates. At 60%-70% of confluency, the cells were harvested and fixed in 70% alcohol for 1-2 h on ice. The cells were treated with RNase A (Sigma) at 37°C for 30 min and stained with propidium iodide (PI) in the dark for 30 min at 4°C. The cell cycle status was assessed by flow cytometry (BD Biosciences, CA, USA) and analyzed using the Cell Quest software.

### BrdU (5-bromodeoxyuridine) incorporation assay

Cells were cultured for 72 h and when they reached 70%-80% of confluency, the cells were treated with 10 μmol/L BrdU for 1 h. After the cells were fixed and their DNA denatured, the incorporated BrdU was detected by APC or PerCP-anti-BrdU (BD Biosciences, Bedford, NY). The cells were analyzed by flow cytometry.

### Clone formation assay

Cells were cultured in 6-cm dish until visible cell colonies were formed. After cells were fixed and stained, the number of cell colonies was counted under a microscope (Leica, Germany).

### Three-dimensional cell culture

Different groups of cells (5000 cells/well) in DMEM/F12 (5% horse serum, 0.5 μg/ml hydrocortisone, 10 μg/ml insulin, 5 ng/ml EGF) containing 2.5% matrigel were cultured in glass-slides (Nunc, Rochester, NY) that had been pre-coated with 100 μl of growth factor-reduced matrigel™ (BD Biosciences). The cells were exposed to fresh medium every four days and imagined on day 8 post culture.

### Immunoprecipitation

Different groups of cells were lysed in buffer containing 50 mM Tris pH 7.2, 1 mM sodium orthovanadate, 50 mM NaF, 25 mM β-glycerophosphate, protease inhibitors, and 1% Triton X-100, and centrifuged. For immunoprecipitation, 10 μg anti-Amot antibodies or 7 μg anti-YAP antibodies were first incubated with 50 μl Dynabeads/protein G (Invitrogen, Carlsbad, CA) for 10 min at room temperature and subsequently reacted with 500 μl cell lysate samples for 30 min at room temperature with gentle rotation to form Dynabead-Ab-Ag complexes. After being washed, the formed immunocomplex was heated for 10 min at 70°C. Finally, the eluted proteins were separated by SDS-PAGE and identified by Western blot analysis using specific antibodies.

### Luciferase reporter assay

786-O and ACHN cells (2000 cells/well) in 96-well plates were co-transfected with 100 ng TEAD-luciferase reporter and 10 ng Renilla expression plasmids using Lipofectamine 2000 (Invitrogen) for 48 hours. The TEAD-luciferase reporter activity was determined using the Dual Luciferase Assay kit, according to the manufacturers’ instruction (Promega, Madison, WI). The firefly luciferase activity of each sample was normalized to that of the internal control Renilla luciferase and the relative luciferase activity was calculated.

### Statistics

Data are representative images or expressed as mean ± SD. The difference among groups was analyzed by ANOVA and student's t-test when applicable using SPSS 13.0 statistical analysis software. A P-value of <0.05 was considered statistically significant.

## SUPPLEMENTARY FIGURE


