# Supplementary material for: Angiomotin promotes renal epithelial and carcinoma cell proliferation by retaining the nuclear YAP
Source: Oncotarget. 2016 Feb 3;7(11):12393–403. doi: 10.18632/oncotarget.7161 (PMC4914293; doi:10.18632/oncotarget.7161)
Supplement: Supplementary file 1 [file oncotarget-07-12393-s001.pdf]

SUPPLEMENTARY FIGURE

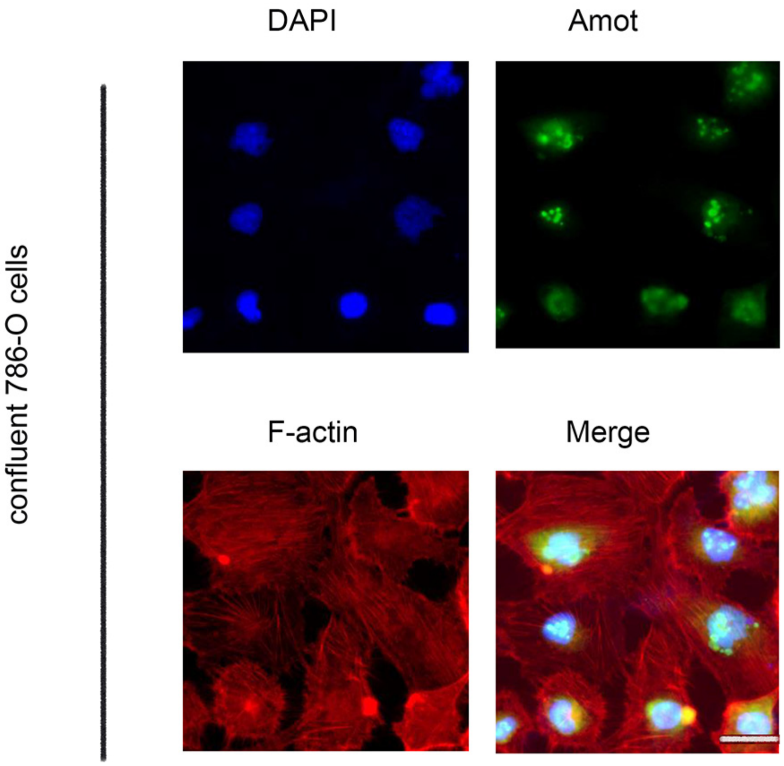

Supplementary Figure S1: Immunofluorescent analysis of Amot and F-actin expression in 786-O cells cultured at confluent density. Scale bar = 25μm.
